# Chicago Veterinary Society

**Published:** 1897-11

**Authors:** James Henderson

**Affiliations:** Secretary


### CHICAGO VETERINARY SOCIETY.

The October meeting was held on the evening of the 14th. It being the annual meeting, the important work of selecting officers for the ensuing year was first for consideration. The work of the officers during the first year being so successful, it was deemed best to continue in office for another term all those who would accept a reëlection. Under this decision the choice of President fell upon the shoulders of Dr. Robert G. Walker; for Treasurer, Dr. G. E. McEvers; Dr. Lawrence Campbell, the very efficient and hard-working Secretary for the first year, declined a reëlection, and the Society, in its appreciation of his faithful services, bestowed on him the honor of first Vice-President. A hearty vote of thanks accompanied this recognition of efficient services. Dr. A. E. Flower was selected as second Vice-President, and Dr. A. Worms as third Vice-President. The choice of Secretary fell upon Dr. James Henderson.

The Committee on Literature and Publication were a target of criticism during the past year for lack of papers for reading. Good results followed the stimulation, and four papers were voluteered for the November meeting.

Dr. Frank Allen introduced the following resolution: "That any of our members who are assistants of the present State Veterinarian (a non-graduate) be invited to appear before our Board of Censors at our next meeting and show cause why their resignations should not be asked for by the Society. This resolution was seconded by Dr. P. Quitman, and carried.

JAMES HENDERSON, M.R.C.V.S.,
Secretary.

